# Porous Chitosan Scaffolds with Embedded Hyaluronic Acid/Chitosan/Plasmid-DNA Nanoparticles Encoding TGF-β1 Induce DNA Controlled Release, Transfected Chondrocytes, and Promoted Cell Proliferation

**DOI:** 10.1371/journal.pone.0069950

**Published:** 2013-07-23

**Authors:** Huading Lu, Lulu Lv, Yuhu Dai, Gang Wu, Huiqing Zhao, Fucheng Zhang

**Affiliations:** 1 Department of Orthopedics, Third Affiliated Hospital of Sun Yat-sen University, Guangzhou, China; 2 School of Materials Science and Engineering, South China University of Technology, Guangzhou, China; 3 Central Laboratory of Third Affiliated Hospital of Sun Yat-sen University, Guangzhou, China; Dowling College, United States of America

## Abstract

Cartilage defects resulting from traumatic injury or degenerative diseases have very limited spontaneous healing ability. Recent progress in tissue engineering and local therapeutic gene delivery systems has led to promising new strategies for successful regeneration of hyaline cartilage. In the present study, tissue engineering and local therapeutic gene delivery systems are combined with the design of a novel gene-activated matrix (GAM) embedded with hybrid hyaluronic acid(HA)/chitosan(CS)/plasmid-DNA nanoparticles encoding transforming growth factor (TGF)-β1. A chitosan scaffold functioned as the three-dimensional carrier for the nanoparticles. Results demonstrated that scaffold-entrapped plasmid DNA was released in a sustained and steady manner over 120 days, and was effectively protected in the HA/CS/pDNA nanoparticles. Culture results demonstrated that chondrocytes grown in the novel GAM were highly proliferative and capable of filling scaffold micropores with cells and extracellular matrix. Confocal laser scanning microscopy indicated that chondrocytes seeded in the GAM expressed exogenous transgenes labeled with green fluorescent protein. ELISA results demonstrated detectable TGF-β1 expression in the supernatant of GAM cultures, which peaked at the sixth day of culture and afterwards showed a moderate decline. Histological results and biochemical assays confirmed promotion of chondrocyte proliferation. Cell culture indicated no affects on phenotypic expression of ECM molecules, such as GAG. The results of this study indicate the suitability of this novel GAM for enhanced *in vitro* cartilage tissue engineering.

## Introduction

Cartilage tissue engineering has been clinically proven to provide better long-term results for treating articular cartilage damage [1]–[3], as compared to the other currently available therapies, such as microfracture, mosaicplasty, and autologous chondrocyte implantation. Moreover, this technique holds particular promise for creating implantable tissue equivalents for orthopedic surgery to overcome the severe shortage of human donors of cartilage tissue. Research continues to enhance the effectiveness of tissue engineering treatments through inclusion of various combinations of growth factors with the aim of inducing, accelerating, and enhancing cartilage formation which more accurately resembles native hyaline cartilage [4].

Transforming growth factor-beta1 (TGF-β1) is an important growth factor in tissue engineering for cartilage repair. TGF-β1 has been shown to promote chondrocyte proliferation and differentiation and increase the synthesis of specific ECMs, both of which are important features of effective cartilage regeneration [4]–[6]. However, the most effective means by which TGF-β1 can be properly introduced in tissue-engineered systems have yet to be fully elucidated. Growth factors have thus far been introduced by: (1) direct addition to the culture medium, (2) overexpression in genetically engineering cells [7], (3) construction of polymeric systems that provide for the controlled release of growth factors [8], (4) direct incorporation of plasmid DNA (pDNA) encoding growth factors into scaffolds [5], [9], and (5) embedding cationic polymeric gene delivery systems that encode growth factors into scaffolds for sustained release of pDNA [10]. Among these, the gene-activated matrices (GAM)-embedded polymeric non-viral gene transfection systems with encapsulated pDNA encoding growth factors appear to have more advantages over the other methods. In this system, the pDNA is more stable and flexible than proteins, and therefore more likely to be compatible with established sustained delivery systems. In addition, As Guo *et al* reported, the GAM gene delivery strategy may protect pDNA from rapid enzymatic degradation and phagocytosis by synovial cells or macrophages in the synovial fluid [5]. Moreover, sustained delivery of pDNA from the GAM polymer matrix aims to transfect a large number of cells within a specific site, resulting in the production of a therapeutic protein and enhancement of tissue development. Therefore, a GAM may serve to enhance the cartilage healing process by effective delivery of growth factors to target tissues [5], [9], [11].

As a natural polysaccharide that is structurally similar to GAGs, chitosan has emerged as a popular agent of cartilage tissue engineering repair systems. Chitosan shows good biocompatibility, biodegradability [12], and capacity to stimulate the activity of growth factors [13]. In addition, it is known to contribute to the maintenance of the chondrogenic phenotype [4], [5]. Chitosan has been widely used as a non-viral gene vector for *in vitro* and *in vivo* gene transfer [14]. Nanoparticles prepared with chitosan and pDNA was proven to be effective in targeted transfection of chondrocytes both *in vitro* and *in vivo* [15], [16]. However, relatively low transfection efficiency has obstructed its further use as an ideal gene transfection vector [17], [18]. Therefore, we have designed hybrid nanoparticles containing hyaluronic acid (HA), chitosan, and pDNA, which showed significantly higher transfection efficiency to chondrocytes than chitosan (CS)/pDNA nanoparticles under the same conditions as in our previous study [19]. As a linear polysaccharide found natively in cartilage, HA functions as a core molecule for the binding of keratin sulfate and chondroitin sulfate in forming aggrecan in cartilage. Moreover, HA included in a scaffold may act as a bioactive molecule, where cell surface receptors for HA (CD44 etc) would allow for cell-scaffold interactions [4], which have been reported to play a crucial role in the development and regeneration of cartilage tissue [20].

In the present study, tissue engineering and local gene delivery strategies are combined in the design of a novel GAM based on a porous chitosan scaffold with embedded hybrid HA/CS/pDNA nanoparticles encoding TGF-β1. The size, zeta potential and morphology of the nanoparticles were characterized using laser diffractometetry and scanning electron microscopy (SEM). The release behavior of the loaded pDNA was monitored over 120 days *in vitro*. *In vitro* expanded chondrocytes were introduced to observe the effect of this novel GAM on chondrocyte adhesion, proliferation, and synthesis of ECMs, and to evaluate the potential efficacy of this novel GAM as a scaffold biomaterial for cartilage tissue engineering.

## Materials and Methods

### Materials

Chitosan (molecular weight 50 kDa, deacetylation degree 90%), chitosanase, and hyaluronidase were purchased from Sigma-Aldrich (St. Louis, MO, USA). Hyaluronic acid, with a molecular weight of 160 kDa, was purchased from C. P. Freda Pharmaceutical Co. Ltd. (Shangdong, China). Dulbecco’s modified Eagle’s medium (DMEM), D-Hanks, and antibiotics were from Gibco (Billings, MT, USA). Fetal bovine serum (FBS) was from Hyclone (Logan, UT, USA). All reagents used were of analytical grade.

### Fabrication of Nanoparticles Loaded with TGF-β1-encoding pDNA

Plasmid DNA contained the enhanced green fluorescent protein expression vector (pEGFP; Invitrogen, Carlsbad, CA, USA) encoding the sequence of TGF-β1 and containing a cytomegalovirus enhancer inserted upstream (pEGFP-TGF-β1). The plasmid was propagated in *Escherichia coli* cells prior to isolation and purification. The absorption ratio was measured at λ = 260 and 280 nm for the evaluation of the plasmid concentration and purity. The same plasmid vector, containing the coding sequence of EGFP but without the insertion of TGF-β1 sequence, was used as an empty plasmid control (pEGFP only).

HA/CS nanoparticles were prepared with incorporated pDNA according to the method described by Duceppe *et al*. [21]. In brief, 10 mg HA was dissolved in 4 mL of buffer (0.1 M sodium acetate and 0.1 M sodium chloride, pH 7.2) with magnetic stirring. After HA was completely dissolved, 0.5 mg hyaluronidase (from bovine testes, activity 300–500 units/mg; Sigma-Aldrich) was added to the solution, which was then kept for 24 hours in a shaking incubator at 37°C. After the enzymatic splitting reaction, the mixture was filtered (Amicon Ultra, MWCO 10 kDa; Millipore Corp, Billerica, MA, USA), and the filtrated HA of low molecular weight (MW<10 kDa) was centrifuged and collected by lyophilization. The resulting substance was dissolved in distilled water (pH 5.5). Ten mg of CS was dissolved in 2% acetic acid (pH 5.5). Both the HA and CS solutions were filtered through 0.22 µm membranes. The CS solution was stirred at a rate of 3000 rpm for 30 min, mixed into the HA solution and stirred for an additional 10 min. Mixtures were prepared with CS:HA weight ratios at 4∶1 (Figure S1). The required volume of 25 µg/mL pDNA was added to the HA/CS solution by gentle pipetting to form complexes of a selected N/P ratio. The N/P ratio was defined as the molar ratio of the positive CS amino group and the negative DNA phosphate group. The resulting mixture was vortexed rapidly for 3–5 s and left for 1 h at room temperature to allow sufficient time for complexes to form completely. Nanoparticle samples were centrifuged at 10,000 rpm for 30 min at 4°C, and were then maintained at −80°C for 24 h, followed by vacuum lyophilization (ALPHA 2–4 LD; Christ, Osterode am Harz, Germany). Lyophilized nanoparticle samples were preserved at 4°C until use.

### Gel Retarding Analysis

In order to assess the degree of protection afforded by HA and CS on incorporated pDNA (encoding EGFP-TGF-β1, 6.5 kbp), either HA/CS/pDNA nanoparticles or naked pDNA were incubated with 4 µg/mL DNase I (Sigma-Aldrich) for 20 min at 37°C. Nanoparticles were also exposed to a 2.78 µg/µL chitosanase (from streptomyces species, activity 18 units/mg; Sigma-Aldrich) digestion for 12 h at 37°C. Naked pDNA, HA/CS/pDNA nanoparticles, and chitosanase digests were investigated by 1% agarose gel electrophoresis.

### Particle Size and Zeta Potential Measurements

A Mastersizer 2000 laser diffractometer (Malvern Instruments, Worcestershire, UK) was used to measure the size, zeta potential, and polydispersity index of the nanoparticles. Nanoparticles were prepared and analyzed in distilled water at 25°C (pH 5.5).

### Fabrication of GAM Embedding HA/CS/pDNA Nanoparticles

CS scaffolds were prepared according to the methods described by Elder *et al*. [22]. Briefly, CS was dissolved in 2% aqueous acetic acid solution to obtain a polymer concentration of 2% (w/v). The CS solution was then poured into a polystyrene 24-well culture plate (2 mL/well), frozen at −60°C, and lyophilized in the ALPHA2-4 LD freeze dryer for 48 h. The lyophilized scaffold was rehydrated in a graded ethanol series from 100% to 70% and stabilized by treatment with 0.5 N NaOH/ethanol mixture (4 v/1 v) for 2 h. Scaffolds were washed with 70% ethanol, and after ethanol evaporation were then rinsed with double-distilled water and were finally lyophilized again for 48 h. The bulk porous cylinders were then trimmed into the final dimensions of 6 mm thickness and 12 mm diameter, washed with double-distilled water and phosphate buffer saline (PBS, pH 7.2), and lyophilized until completely dry. Then, the scaffolds were cross-linked by 1-ethyl-3-(3-dimethyl aminopropyl)carbodiimide (EDC, 0.5% (w/v), 3 ml for each sample), as described previously [23]. Ethylene oxide was used to sterilize scaffolds. A 400 µL HA/CS/pDNA nanoparticles suspension (the concentration of pDNA encoding EGFP-TGF-β1 was 25 µg/mL, and the final pDNA dose was 10 µg for each scaffold) was then dropped onto the dried scaffold shortly after preparation, and maintained at 4°C overnight for full incorporation. A control scaffold was generated by dropping a 400 µL HA/CS/pDNA nanoparticles suspension (the concentration of pDNA encoding EGFP was 25 µg/mL, and the final pDNA dose was 10 µg for each scaffold). These GAMs were then frozen at −70°C for 2 h and lyophilized until completely dry for pDNA release and cell culture studies. A porous CS scaffold containing no HA/CS/pDNA nanoparticles served as a second control. The degree of open porosity of scaffolds was estimated using the liquid displacement method [24].

### *In vitro* pDNA Release Studies

The quantity of pDNA release from HA/CS/pDNA nanoparticles and GAM-nanoparticle composites was determined by incubating the nanoparticles or GAM-nanoparticle composite in PBS at 37°C in a shaker bath at 100 rpm. At appropriate time intervals (6 h, 12 h, 24 h, 3 d, 5 d, 7 d, 10 d, 15 d, 20 d, … 120 d), samples were centrifuged at 13,200 rpm for 15 min and the supernatants were collected for quantification and replaced with equal volumes of fresh PBS solution [25]. The amount of pDNA released into the supernatant was measured spectrophotometrically at 260 nm (DU640; Beckman, Fullerton, CA, USA). The supernatant of empty (no DNA) HA/CS nanoparticles was used to normalize the absorption at 260 nm. After incubation and precipitation with ethanol, samples were checked with agarose gel electrophoresis as described above. Each type of sample was tested in duplicate (*n* = 6).

### Chondrocyte Isolation and Culture with Scaffolds *in vitro*

All procedures involving animals in this study were reviewed and approved by the Institutional Animal Care and Use Committee at the Sun Yat-sen University Guangzhou, China (Approval ID: 2010-0401). Cartilage tissue was harvested under sterile conditions from the knee joints of three-week old New Zealand white rabbits (*n* = 12; Laboratory Animal Center of Sun Yat-Sen University, Guangzhou, China. Animal quality certificate numbers: 0064121, 0069181, and 0069646). Briefly, articular cartilage was sliced and minced into small pieces (1–2 mm^3^ volume), digested in 0.25% trypsin (Gibco) for 30 min, thoroughly washed and incubated at 37°C for 12 h with 0.2% (w/v) collagenase II (activity 277.0 units/mg; Gibco). After digestion with trypsin and collagenase II, the tissue fragment had dissolved; and cells were harvested every 2 h, for a total of 4–6 times. After centrifugation at 1000 rpm for 10 min, cells were collected and seeded in flasks at a density of 2×10^5^ cells/mL in DMEM supplemented with 10% FBS, streptomycin at 100 µg/mL, and penicillin at 100 U/mL. After four days of culturing at 37°C with 5% CO_2_ in a humidified atmosphere, ∼90% confluence had been reached and chondrocytes were digested using 0.25% trypsin and resuspended in fresh culture medium at a cell density of 2×10^7^ cells/mL. Sterilized scaffolds were transferred into 24-well plastic culture plates (one scaffold per well). The scaffolds had been pre-wetted with culture medium and incubated overnight to allow for gas exchange, after which they were dried by sterilized filter paper. Two-hundred µL of cell suspension was seeded onto each scaffold (4×10^6^ cells/scaffold). After 2 h, another 1.8 mL of culture medium was added, and the resulting culture was incubated at 37°C in a humidified chamber with 5% CO_2_. Culture medium was changed every 2–3 days.

### Scanning Electron Microscopy Examination

The CS scaffolds with or without embedded HA/CS/pDNA nanoparticles were examined by SEM (JSM-6330; JEOL, Tokyo, Japan) to evaluate their porosity.

Chondrocyte-seeded scaffolds were also examined using SEM to observe cell morphologies. Samples incubated for 7 days *in vitro* were washed twice with sterilized PBS, fixed in 2.5% (v/v) glutaraldehyde for 12 h, followed by dehydration through a graded series of ethanol for 15 min at each ethanol concentration, and lyophilized for 16 h. The samples were finally sputter-coated with gold–palladium film for SEM observation.

The morphology of HA/CS/pDNA nanoparticles was also evaluated by SEM. Previously lyophilized nanoparticles were dissolved in distilled water and dispersed with ultrasound to create a suspension. The HA/CS/pDNA nanoparticle suspension was dropped onto a clean glass slide and dried at 37°C overnight to evaporate the water. The slide was then coated with gold and observed by SEM.

### Confocal laser Scanning Microscope Observation

Sterilized scaffolds were shaped into cylinders of 0.8 mm thickness and 12 mm diameter (with or without 4 µg initial pDNA), placed into cell culture dishes (Corning, NY, USA), and seeded with 2×10^5^ chondrocytes and cultured as described above. Chondrocyte-scaffold complexes were examined with a confocal laser scanning microscope (LSM710; Zeiss, Jena, Germany) on 7, 14, and 21 days after seeding to determine if chondrocytes could be efficiently modified to express exogenous transgenes.

### Enzyme-linked Immunosorbent Assay (ELISA)

The amount of TGF-β1 secreted into the culture medium was determined after 3, 6, 9, 12, 15, 18, and 21 days of culture using a commercial TGF-β1 ELISA kit (Bender MedSystems GmbH, Vienna, Austria) according to the manufacturer’s instructions. Chondrocyte-seeded scaffolds (with 4×10^6^ initial implant cells, with or without 10 µg initial pDNA) were incubated at 37°C, with medium change every three days. Three samples were collected from each group for each time point for ELISA analysis. Cell-seeded CS scaffolds of DNA-free and cell-seeded pEGFP-activated scaffolds were used as control groups. The mean concentrations of TGF-β1 (the instantaneous concentration at that time) were compared by repeated measurement ANOVA (*n* = 3). A *p*-value of <0.05 was considered to be significant.

### Histological and Immunohistochemical (IHC) Analyses

Cell-seeded scaffolds (with 4×10^6^ initial implant cells, with or without 10 µg initial pDNA) were evaluated by hematoxylin & eosin (H&E) and toluidine blue staining after 7 or 21 days of culture. Samples were fixed in 10% (v/v) buffered formaldehyde at 4°C, dehydrated in 90% and 100% ethanol twice for 30 min each, and embedded in paraffin. The paraffin-embeded samples were cut into sections 5 µm in thickness, stained with H&E and toluidine blue, and visualized using a light microscope. IHC staining was used to detect collagen II secretion. Slides were deparaffinized, washed with PBS, digested with 1% trypsin for 30 min, and then pretreated with 5% bovine serum albumin (BSA; Boster Biological Technology Co., Ltd., Wuhan, China) at 37°C for 15 min to block nonspecific reactions. Samples were then incubated with 1∶100 diluted mouse anti-rabbit primary antibody of collagen II (Calbiochem, Merck, Darmstadt, Germany) at 4°C overnight, washed with PBS, incubated with biotinylated goat anti-mouse secondary antibody (Boster) at 37°C for 20 min, washed with PBS, and reacted with Streptavidin–Biotin Complex (SABC; Boster) at 37°C for 20 min. Finally, diaminobenzidine (DAB; Boster) was used as the chromogen. All sections were counterstained with hematoxylin. Tissue sections stained without primary antibodies were used as negative controls.

### Biochemical Assay Detection of Chondrocyte Proliferation and GAG Production

After several interval days (3 d, 7 d, 14 d, 21 d) of incubation, four pieces of the co-cultured samples (with 4×10^6^ initial implant cells, with or without 10 µg initial pDNA) were taken from the three formulations. The samples were rinsed in 2.5 mL PBS. The DNA content of the matrices was evaluated for each using the Hoechst 33258 fluorescent dye assay, as described previously [26]. Briefly, the cell-seeded matrices were digested in a 2.5 ml solution containing 0.2 M NaCl, 0.1 M NaAc, 0.01 M L-cysteine-HCl, 0.05 M EDTA-Na_2_ (pH 6.0), and 2 U papain for 18 h at 65°C. A 200 µL aliquot was analyzed for total DNA by the addition of 100 µL Hoechst solutions (0.2 µg Hoechst 33258/mL). The fluorescence of the samples was measured at an excitation of 365 nm and an emission of 458 nm. Calf thymus DNA was used as the standard.

The GAG content of the matrices was assessed by modification of the dimethylene blue method [27]. Aliquots (0.5 mL) of the papain digest were assayed for GAG after the addition of 2.5 mL l, 9-dimethylmethylene blue dye (DMMB) solution. The absorbance at 535 nm was measured with a spectrophotometer. To obtain the amount of GAG per sponge, the results were extrapolated from a standard curve using chondroitin sulfate.

For GAG and DNA analyses, the values of the unseeded controls of the three formulations (*n* = 4) were subtracted from the seeded samples, and the cell-seeded DNA-containing scaffold was corrected with the unseeded controls having the same amount of DNA in the scaffold. The DNA dosage was calculated by subtracting the concentration of DNA released in the culture medium from the original concentration of DNA used in the analysis.

### Statistical Analysis

All data are presented as mean ± standard deviations (s.d.) and, when needed, analyzed using ANOVA for repeated measurement design by using the statistical software package SPSS v13.0. A *p*-value of <0.05 was considered to be significant.

## Results

### Characterization of HA/CS/pDNA Nanoparticles

**Figure 1:**
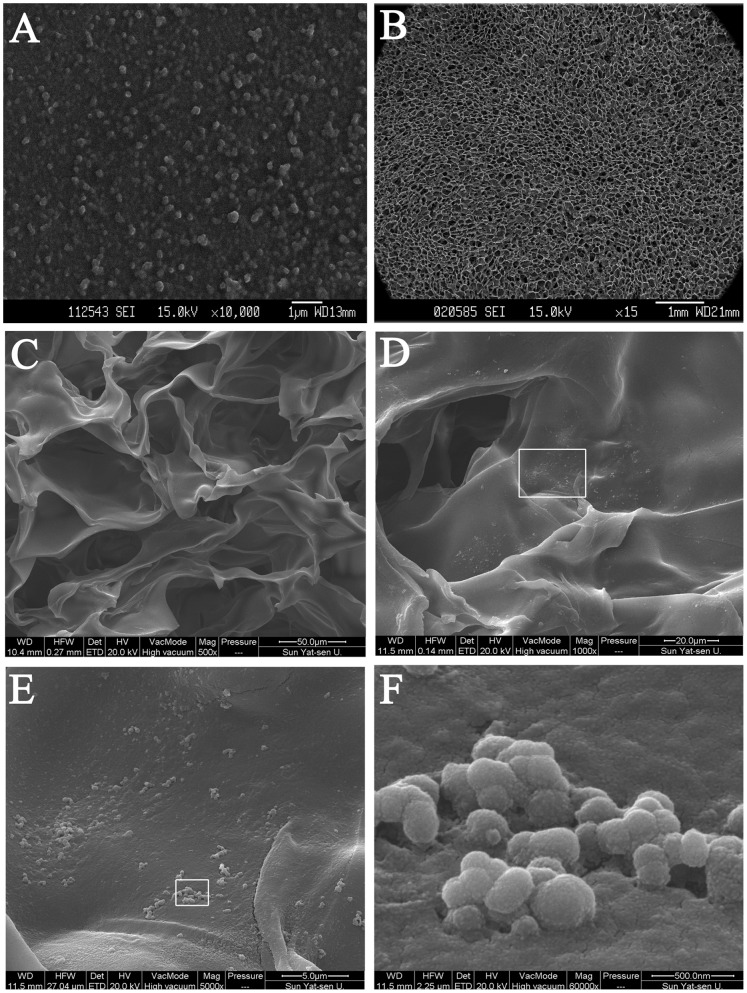
Scanning electron microscopy (SEM) micrographs of nanoparticles and scaffolds. Panel A shows a representative image of lyophilized HA/CS/pDNA nanoparticles prepared with a weight ratio of CS:HA of 4∶1 and 25 µg/mL of pDNA. Panel B shows the porous chitosan scaffolds without nanoparticles. Panel C shows a GAM scaffold embedding HA/CS/pDNA nanoparticles. Panel D–F shows a gradually magnified view of GAM surface morphology featuring entrapped HA/CS/pDNA nanoparticles. Panel F shows many spherical nanoparticles on the surface of the scaffold, ranging in size from 100–300 nm.

SEM images of HA/chitosan/pDNA nanoparticles showed an even distribution of particles, most of which had a regular spherical shape and a diameter of 100–300 nm (Figure 1A). Using laser diffractometetry, the size of the HA/chitosan/pDNA nanoparticles was found to be 155.4±28.4 nm, the zeta potential 22.14±1.92 mV, and the polydispersity index 0.234±0.013 (pH = 5.5).

### Electrophoresis Assay

**Figure 2:**
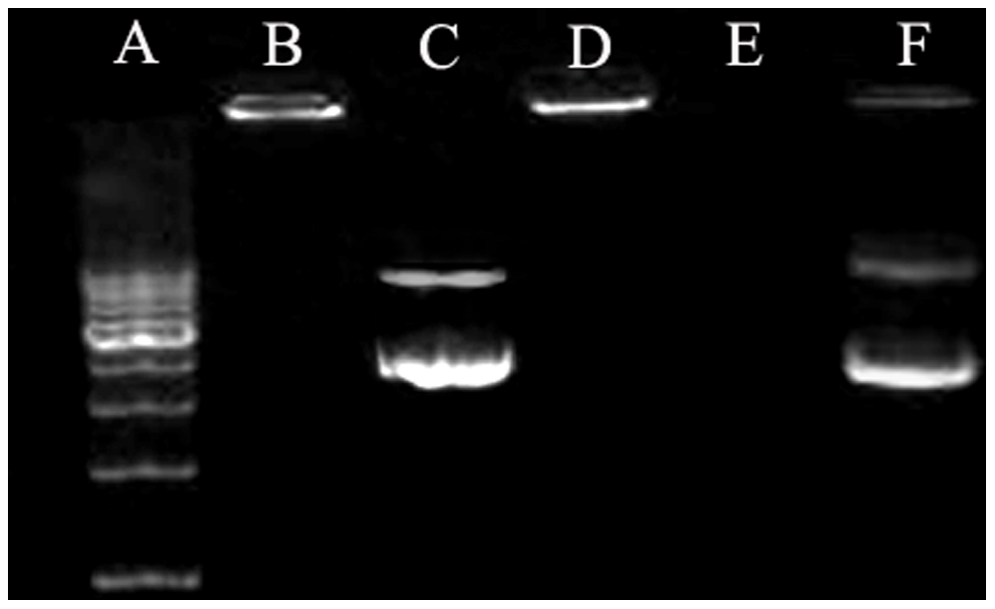
Gel electrophoresis of HA/CS/pTGF-β1 nanoparticles, digests, and control samples. Lane A shows the DNA molecular weight marker. Lane B shows HA/CS/pTGF-β1 nanoparticles. Lane C shows normal naked pTGF-β1. Lane D shows HA/CS/pTGF-β1 nanoparticles digested by DNase I. Lane E shows naked pTGF-β1 digested by DNase I. Lane F shows HA/CS/pTGF-β1 nanoparticles digested by chitosanase (2.78 µg/µL).

Agarose gel electrophoresis assay indicated pDNA was exceptionally well protected by HA/CS/pDNA nanoparticles and maintained its integrity (Figure 2). In Figure 2, lane B demonstrates that pDNA was totally retained within the gel-loading well, which illustrates the complete combination of pDNA with cationic chitosan. After adding DNase I, the fluorescent light originating from the lane containing naked pDNA disappeared completely (Figure 2, lane E), whereas the fluorescence intensity of lanes containing HA/CS/pDNA nanoparticles decreased only slightly (Figure 2, lane D). This indicates that the pDNA within HA/CS/pDNA nanoparticles was subject only to slight nuclease degradation or to no degradation at all. These results indicate that HA/chitosan/pDNA nanoparticles entrap DNA. However, after treatment by chitosanase, degradation of the chitosan led to release of the entrapped pDNA from the nanoparticles and consequent migration into the gel (Figure 2, lane F).

### The pDNA Release Curve of HA/CS/pDNA and GAM

**Figure 3:**
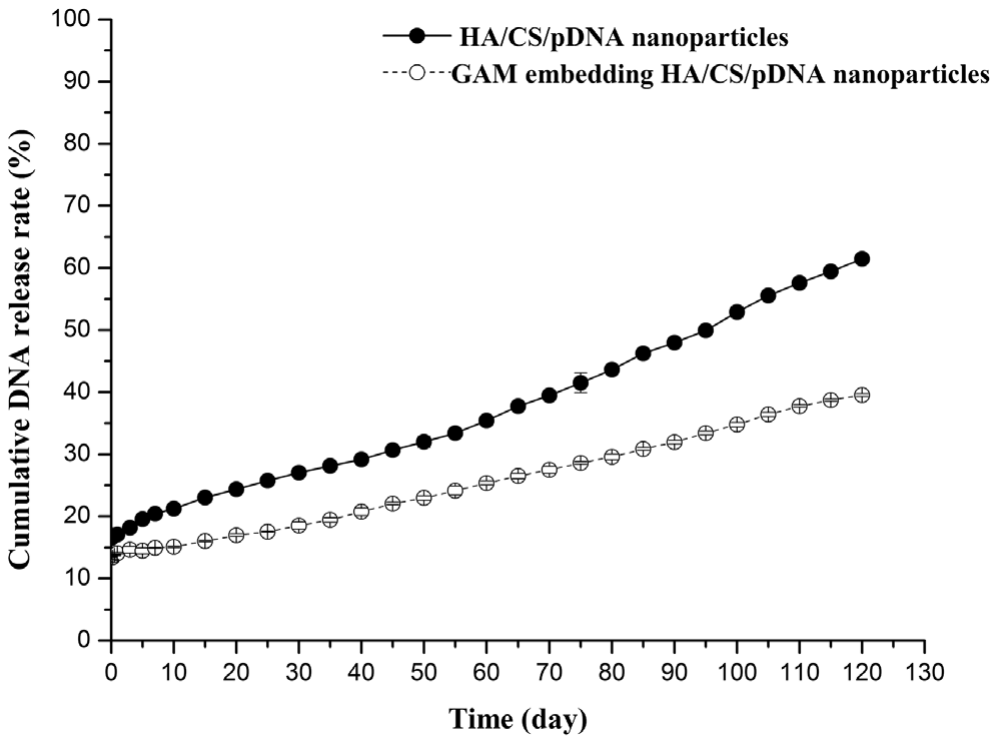
Cumulative pDNA release profiles of HA/CS/pDNA nanoparticles and the GAM embedding HA/CS/pDNA nanoparticles as a function of time up to 120 days.

The pDNA release profiles of the HA/CS/pDNA nanoparticles and GAM embedded with HA/CS/pDNA nanoparticles both exhibit a small burst of pDNA release of about 10–20% in the first 24 h, followed by a slow release at a constant rate (Figure 3). As shown in Figure 3, the pDNA release from nanoparticle-embedded GAM remained steady with a very slow increase after 24 h, reaching approximately 40% (39.21±0.22%) 120 days later. However, the pDNA release characteristic of the HA/chitosan/pDNA nanoparticles was much higher than that of GAM, reaching more than 60% (61.47±0.59%) 120 days later.

### Scaffold Morphology

Chitosan scaffolds exhibited an interconnected, highly porous microstructure (86.0±1.2% porosity) with pores ranging in size from 100–300 µm (Figure 1B). With introduction of HA/CS/pDNA nanoparticles to the chitosan scaffolds, pore size did not change significantly. In scaffolds embedding HA/CS/pDNA nanoparticles, clusters of nanoparticles were observed as distributed along the pore wall of scaffolds (Figure 1C–F).

**Figure 4:**
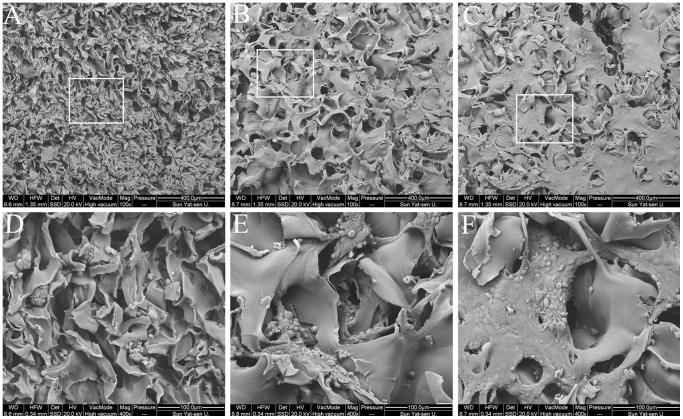
SEM images of chondrocytes cultured for 7 days with plain chitosan scaffolds (A and D), GAM scaffolds embedding empty plasmid scaffolds (B and E), and GAM scaffolds embedding TGF-β1 nanoparticles (C and F). Chondrocytes formed confluent cells on the surface of GAM scaffolds. Panels A, B, C: ×100; D, E, F: ×400.

The scaffolds expanded once placed in media, creating pores that were large enough for nutrients to pass through. Figure 4 shows SEM micrographs of chondrocytes cultured with the three different kinds of scaffolds after 7 days of culture. Chondrocytes adhered well onto surface of the scaffold or penetrated the scaffold along the pores. In some places, chondrocytes proliferated and grew together to form confluent cells, which indicates that GAM displayed good cytocompatibility.

### Confocal Laser Scanning Microscope Observation

**Figure 5:**
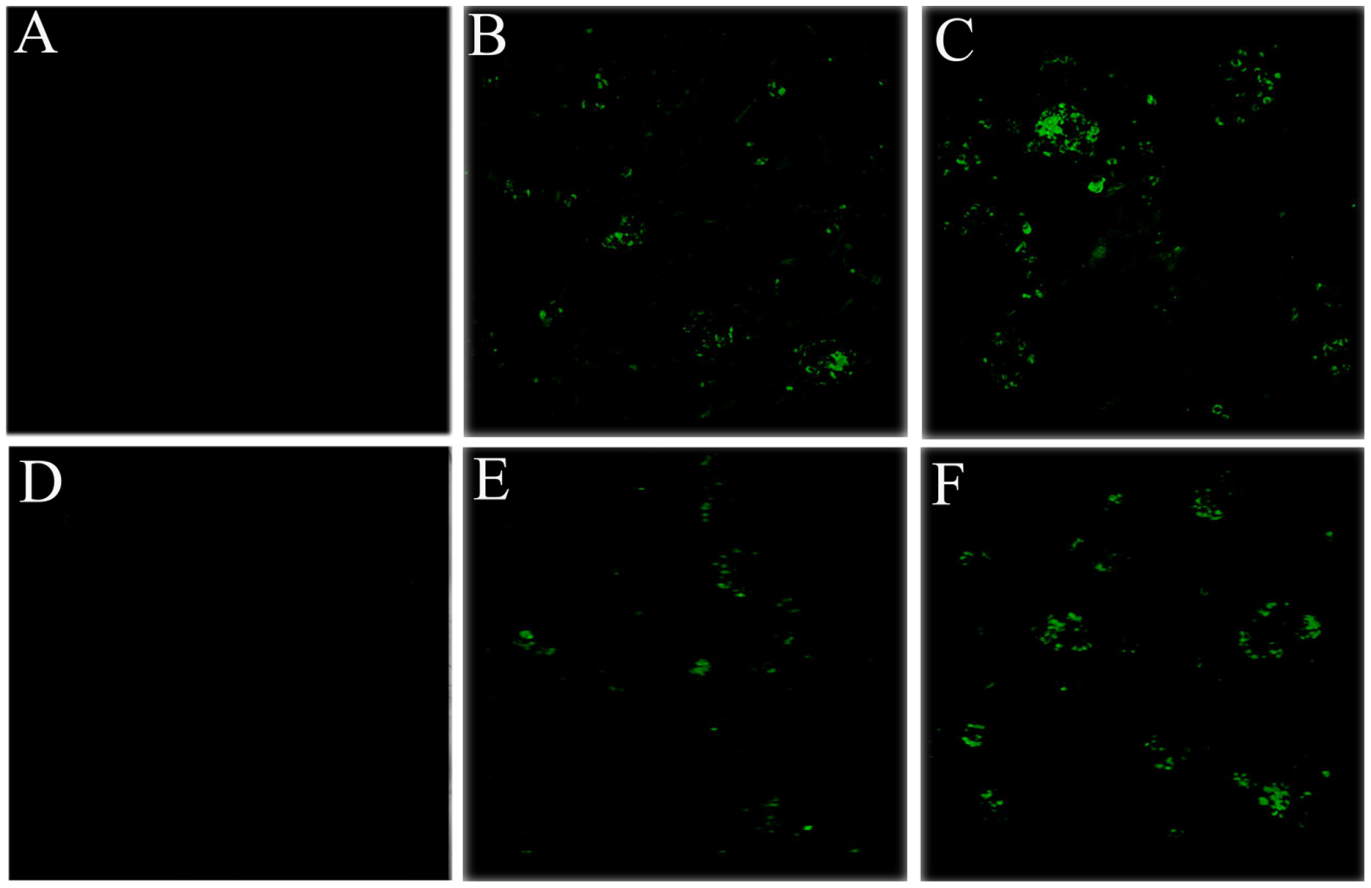
Confocal laser scanning microscopic observation of chondrocyte-scaffolds cultures on the 7^th^ and 21^st^ day. Panels A, B, C show chondrocytes cultured for 7 days while panels D, E, F show chondrocytes cultured for 21 days. A large number of GFP positive chondrocytes clusters are visible for culture with GAM embedding HA/CS/pDNA nanoparticles encoding either EGFP (B and E) or TGF-β1(C and F), indicating transfection by expression of GFP. No GFP-positive cells were detected in chitosan-only scaffolds (A and C). Images come from screenshots of 3-D scanning images of the examined area.

Confocal laser scanning microscopy was used to observe the GFP to determine transfection efficiency of chondrocytes *via* the nanoparticle embedded scaffold (GAM). Transfection efficiencies were assessed by GFP on days 7, 14, and 21 after cell culture. On the 7^th^ day, a large mass of chondrocytes in GAM embedding HA/CS/pDNA nanoparticles encoding EGFP or EGFP-TGF-β1 appeared to express GFP, while no GFP-positive cells were detected in the chitosan scaffold free of DNA and nanoparticles (Video S1 and Video S2). The fluorescence intensity in the pTGF-β1-activated GAM was as strong as that in the pEGFP-activated GAM. On the 14^th^ day, cell clusters in both types of GAM still expressed GFP. On the 21^st^ day, the GFP expression level in both types of GAM was observed to decrease to some degree (Figure 5, Video S3 and Video S4). In addition, an experiment using confocal microscopy to assess FITC-labeled DNA and DAPI-stained nuclei showed that DNA of HA/CS/pDNA nanoparticles entered into the chondrocytes’ nuclear compartment after 4 h of incubation (Figure S2).

### TGF-β1 Expression

**Figure 6:**
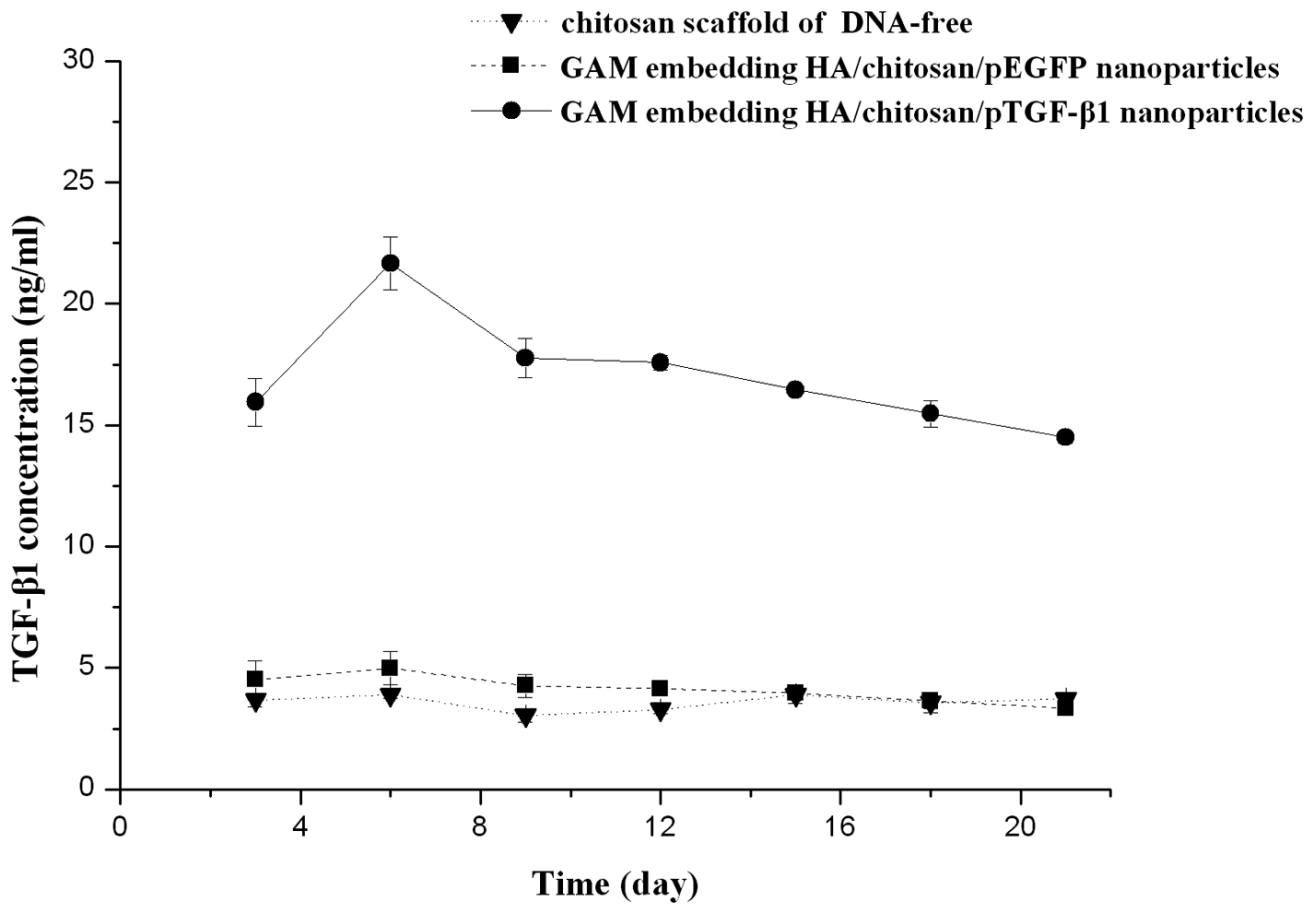
The TGF-β1 expressed in cultures was analyzed by ELISA. Chondrocytes incubated in the GAM embedding HA/CS/pTGF-β1 nanoparticles produced a higher level of TGF-β1 during the culture period. The maximum concentration of TGF-β1 in the culture media was detected on day 6, after which point levels declined moderately (*n* = 3).

For ELISA assay of TGF-β1 expression, ANOVA of repeated measurement design was used to compare the mean concentrations of TGF-β1 of different group. Sphericity assumption was not valid by the test (*p = *0.031), we used the Greenhouse-Geisser test to perform the data analysis. Chondrocytes cultured with the GAM embedding the HA/CS/pTGF-β1 nanoparticles produced higher levels of TGF-β1 during the culture period (*F = *2913.003, *p*<0.001). TGF-β1 in the supernatant could be clearly detected after three days of culture, reaching the maximum concentration on the sixth day, and then followed by a moderate decline. These results were consistent with the expression of GFP detected by confocal laser scanning microscope observation. The peak concentration of TGF-β1 was 22.0±1.1 ng/mL in chondrocytes cultured with GAM embedding HA/CS/pTGF-β1 nanoparticles. Twenty-one days after incubation, TGF-β1 was still maintained at a high level (*F = *54.375, *p*<0.001, Figure 6).

### Histology

**Figure 7:**
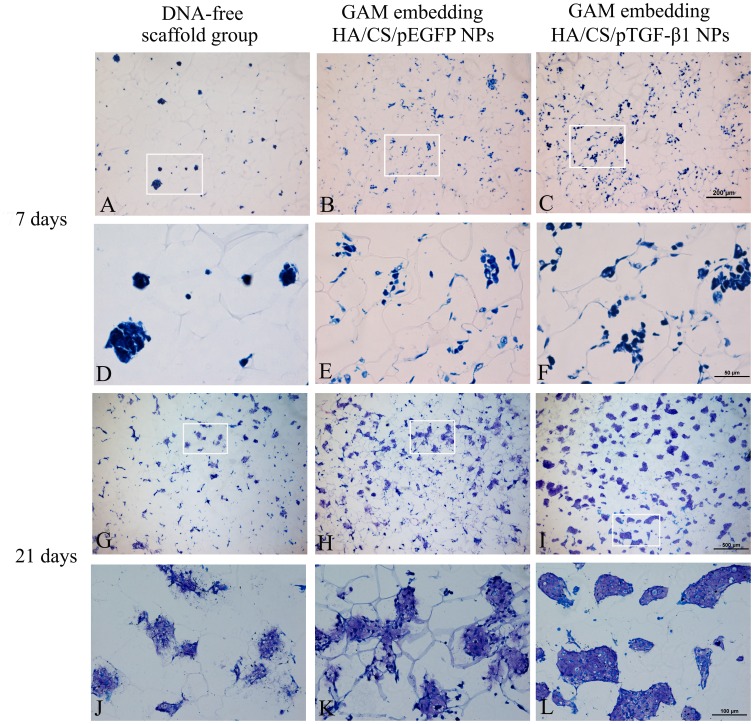
Histology (toluidine blue staining) of chondrocytes seeded to chitosan-only scaffolds, GAM embedding empty plasmid nanoparticles, and GAM embedding HA/CS/pTGF-β1 nanoparticles for 7 and 21 days of culture. Toluidine blue stains ECM, therefore cell clusters intensely stained by toluidine blue indicate newly synthesized proteoglycans. Cell clusters were much denser and evenly distributed in GAM embedding HA/CS/pTGF-β1 nanoparticles than the other two control groups. Panels A, B, C: ×100; D, E, F: ×400; G, H, I: ×40; J, K, L: ×200.

The attachment and growth of primary chondrocytes cultured with scaffolds was observed by H&E staining. In the first week of culture, for the chitosan scaffold of the nanoparticle and DNA-free group, cells were found loosely dispersed in the scaffolds. Chondrocytes maintained a spherical morphology and attached onto the scaffold surface and penetrated into the scaffold interior through pores. In contrast to chondrocytes grown on the nanoparticle-free chitosan scaffold, at 7 days a significant increase in cell number was observed for chondrocytes cultured in the two GAMs embedding nanoparticles, especially in the pTGF-β1 activated GAM. Interestingly, most chondrocytes had merged to form large scaffold-adherent aggregates in the HA/CS/pTGF-β1 nanoparticle-embedded GAM, which demonstrated higher cell density (Figure S3). Figure 7 shows that the cell clusters in the HA/CS/pTGF-β1 nanoparticle embedded GAM were surrounded by ECM that was more intensely stained by toluidine blue, which identifies these regions as containing newly synthesized proteoglycans.

When cultured for 21 days, remarkable differences appeared between the three groups. For GAM embedding HA/CS/pTGF-β1 nanoparticles, the density of cells increased significantly and formed clusters in the scaffold micropores at a much higher cell density than the other two scaffold types. Most cells maintained their characteristic spherical shape, proliferated, and were surrounded by a large amount of newly synthesized pericellular matrix. For the GAM embedding HA/CS/pEGFP nanoparticles, a much denser, even distribution of cell clusters was observed, as compared to the chitosan-only scaffold. Figure 7 shows cell clusters surrounded by ECM stained intensely by toluidine blue. On the basis of these observations of the cell-scaffold constructs after 21 days of culture, a significant proliferation of chondrocytes is indicated since most open pores of the scaffold were occupied by a number of cell aggregates composed of chondrocytes and newly synthesized pericellular matrix. This was especially the case for GAM embedding HA/CS/pTGF-β1 nanoparticles.

### Type II Collagen Levels

**Figure 8:**
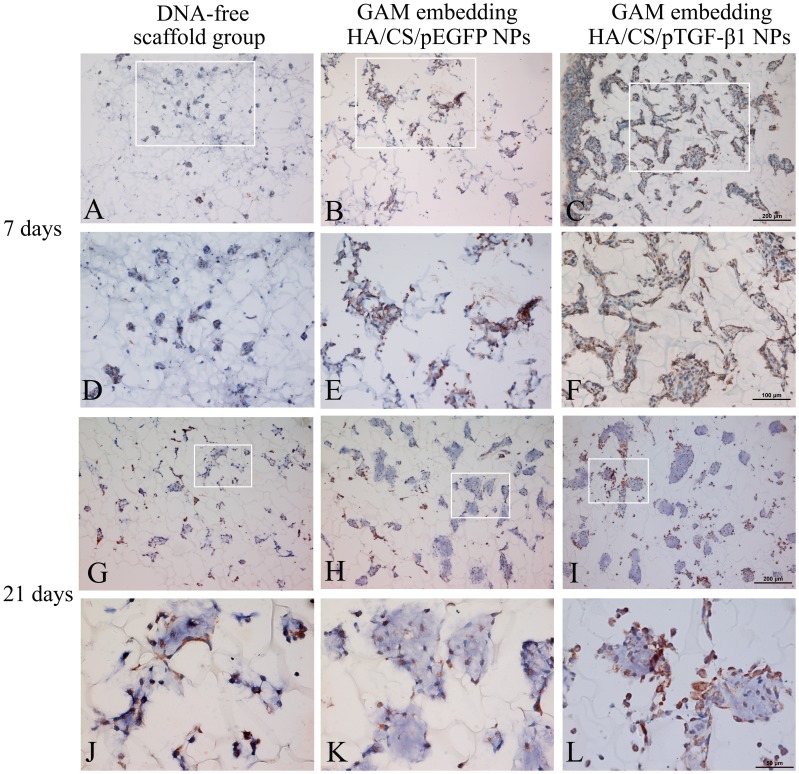
Immunohistochemistry staining for type II collagen in chondrocytes cultured on the chitosan-only scaffold, GAM embedding HA/CS/pEGFP nanoparticles, and GAM embedding HA/CS/pTGF-β1 nanoparticles for 7 and 21 days. Panels A, B, C: ×100; D, E, F: ×200; G, H, I: ×100; J, K, L: ×400.

Type II collagen is the most important marker of chondrogenesis, as it is the major component of cartilage-specific ECM. Therefore, IHC staining was used to assess levels of type II collagen. Figure 8 shows type II collagen at 7 days of culture and 21 days of culture. As shown in Figure 8, type II collagen was strongly stained both intracellularly and extracellularly in the GAM embedded with HA/CS/pTGF-β1 nanoparticles, indicating higher production of type II collagen in this group. In the control groups, weakened expression of type II collagen was indicated, especially in the chitosan scaffold free of nanoparticles. After 21 days, type II collagen remained strongly stained in cell clusters of the TGF-β1 activated GAM scaffold. However, chondrocytes cultured in controlled scaffolds, especially the chitosan scaffold without nanoparticles, showed weaker and more limited expression of type II collagen.

### DNA and Glycosaminoglycan Assay

**Figure 9:**
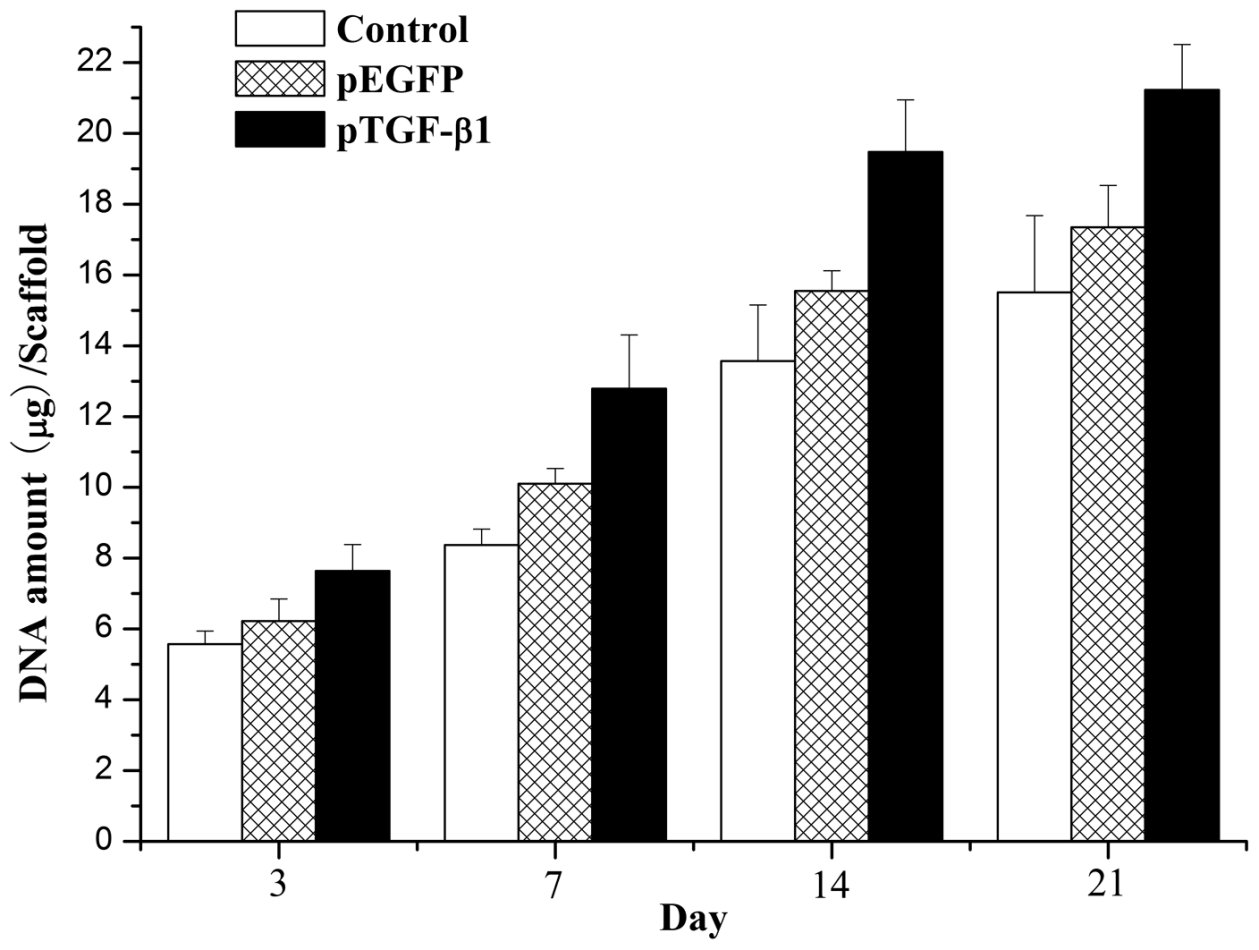
DNA content per sample for chitosan-only scaffolds encapsulating chondrocytes, chondrocytes/GAM embedding HA/CS/pEGFP nanoparticles, or chondrocytes/GAM embedding HA/CS/pTGF-β1 nanoparticles. At a given time point, samples marked by (*) exhibited significantly higher cellularity than chitosan scaffolds of DNA-free samples containing only chondrocytes. Samples indicated with (**) had significantly higher DNA amount than the other two groups (*p*<0.05). Error bars represent mean (*n* = 4) ± s.d.

Proliferation of and extra-cellular matrix production by chondrocytes were assessed by determining the DNA content and GAG content at days 3, 7, 14 and 21 of culture with the three construct formulations. (For DNA content assay, Sphericity assumption was valid by the test (*p = *0.409) and the ANOVA for repeated measurements was used to analyze the data) As shown in Figure 9, all matrices revealed a gradual increase in the total amount of DNA during the culture period from day 3 to day 21, indicating proliferation of chondrocytes (*F = *305.403, *p*<0.001). When compared to the cellularity of DNA-free constructs (group 1), significantly higher cellularity was observed at day 7 and beyond for GAMs with HA/CS/pDNA nanoparticles loading (with or without TGF-β1 loading). This difference was even more obvious for GAMs encapsulating the TGF-β1-loaded HA/CS/pDNA nanoparticles (*p*<0.001).

**Figure 10:**
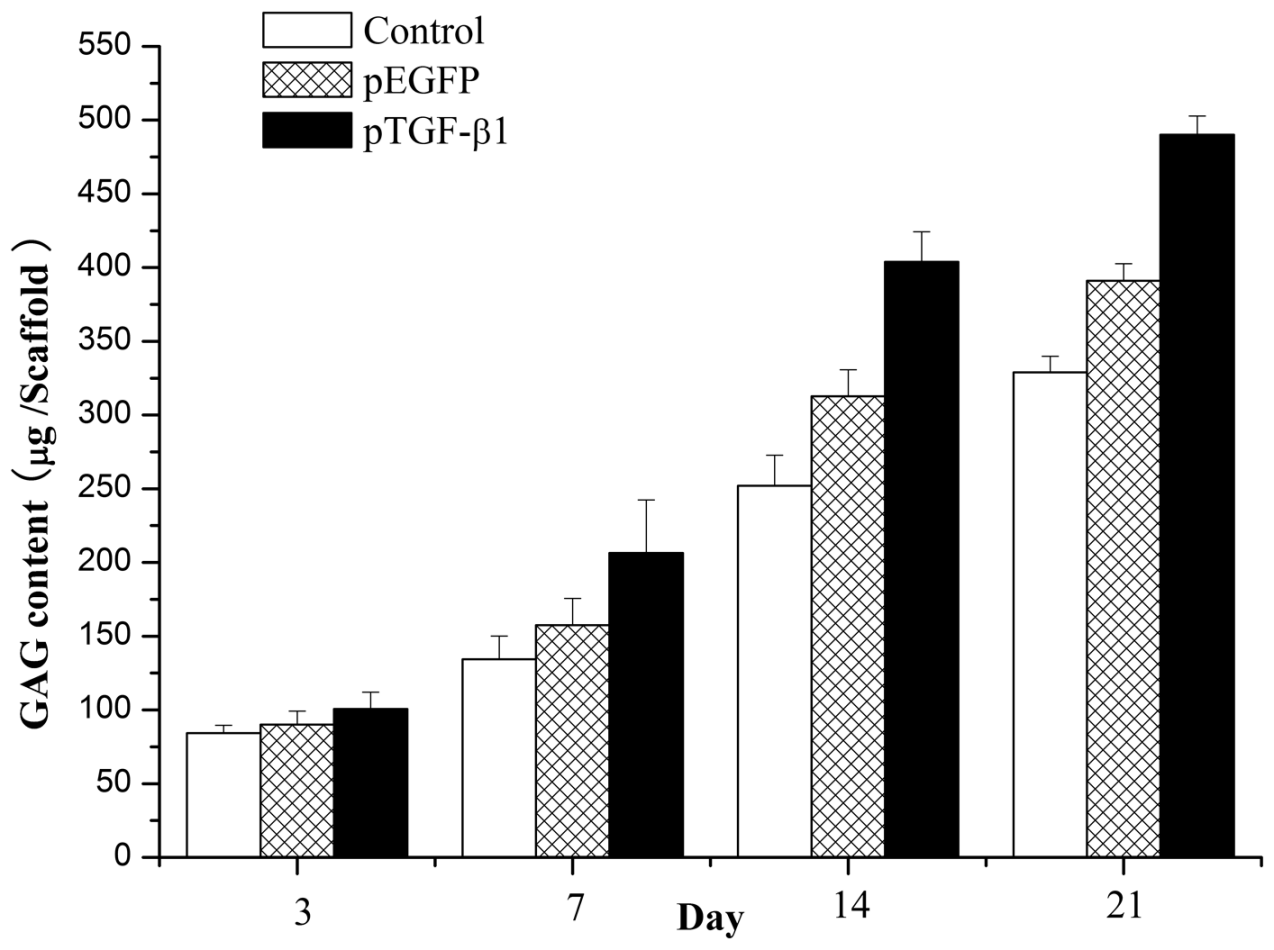
GAG content per sample for chitosan-only scaffolds encapsulating chondrocytes, chondrocytes/GAM embedding HA/CS/pEGFP nanoparticles, or chondrocytes/GAM embedding HA/CS/pTGF-β1 nanoparticles. At a given time point, (*) indicates that samples of chondrocytes/GAM embedding HA/CS/pTGF-β1 nanoparticles exhibit significant higher (*p*<0.05) GAG than samples of the other two group. Error bars represent mean (*n = *4) ± s.d.

As shown in Figure 10, all groups demonstrated an increase in GAG production per construct over the 21-day culture period (*F = *2220.882, *p*<0.001). By day 14, constructs encapsulating unloaded HA/CS/pDNA nanoparticles and TGF-β1-loaded HA/CS/pDNA nanoparticles possessed significantly higher GAG content than those measured at day 3 of culture. At day 21, the GAG content per sample was significantly higher in constructs with HA/CS/pDNA nanoparticles, as compared to constructs without nanoparticles (*p*<0.001). Furthermore, GAG content in constructs with TGF-β1-loaded HA/CS/pDNA nanoparticles remained significantly higher for the remainder of the culture period (*p*<0.001).

**Figure 11:**
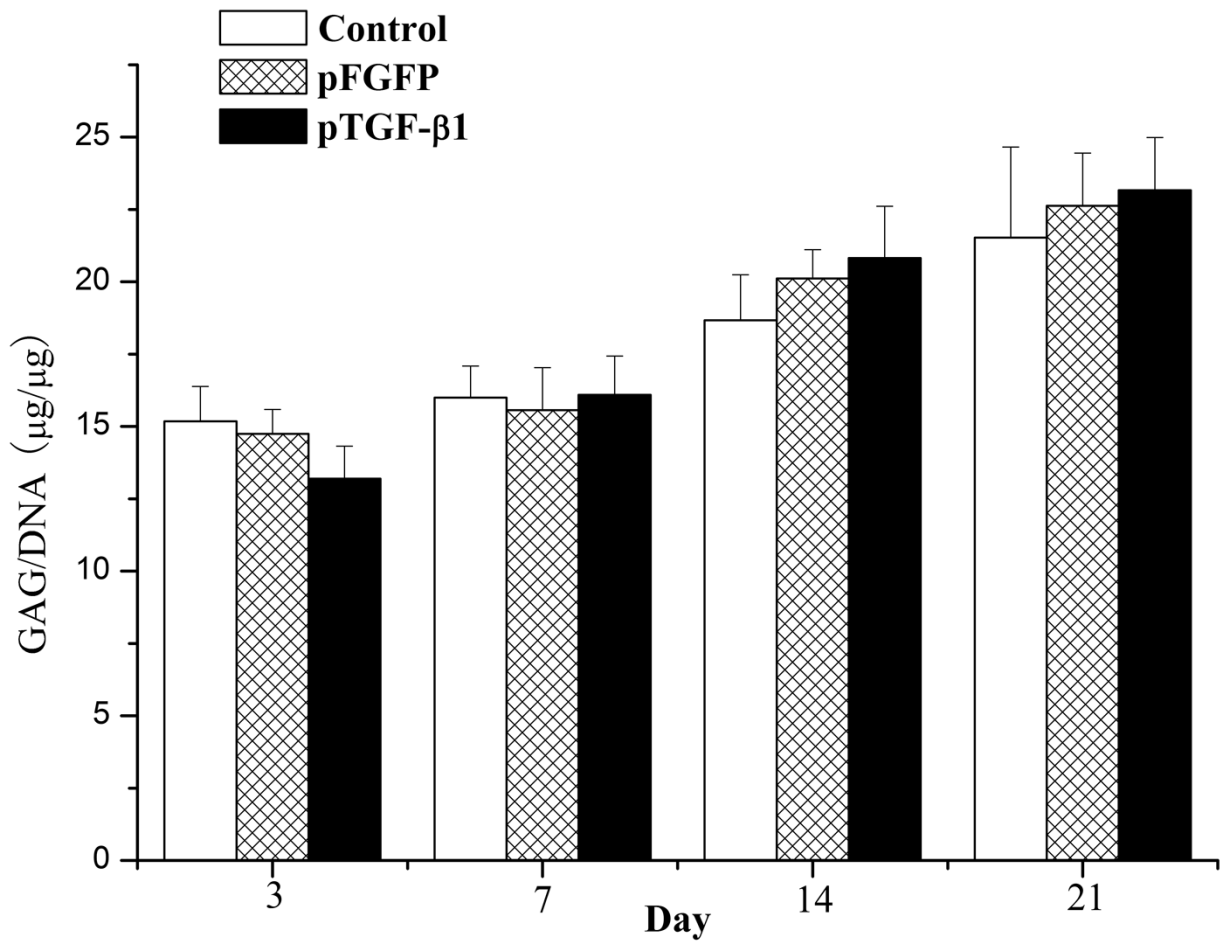
GAG content per amount of DNA of chondrocyte in chitosan-only scaffolds, GAM embedding HA/CS/pEGFP nanoparticles, or GAM embedding HA/CS/pTGF-β1 nanoparticles. No significant differences were observed when values were compared amongst experimental groups at a given time point. Error bars represent mean (n = 4) ± s.d.

In Figure 11, GAG content is normalized to cellular DNA content measured for each matrices construct. No significant differences in values of GAG per µg of cellular DNA were observed when comparing among formulations at any given time point (ANOVA for repeated measurements was used as the sphericity test was identified by the test *p = *0.252, multiple comparison tests, *p*>0.05).

## Discussion

The application of growth factors, such as TGF-β1, can enhance synthesis of articular cartilage, which is required since cartilage lacks a blood supply and thus receives limited amounts of nutritional substances [5]. However, a high dose of TGF-β1 *via* intra-articular injection is known to induce chemotaxis and activation of inflammatory cells, resulting in characteristic cartilage defects such as fibrosis and osteophyte formation [22], [24], [28]. It is evident that TGF-β1 should be administered in a controlled manner to minimize adverse effects. Gene therapy may be an ideal technique to deliver this short-lived cytokine [29]–[31], as it offers the advantages of inducing local expression and increased DNA stability. Incorporation of DNA into tissue engineering scaffolds, and its subsequent sustained release, may provide an optimal means to engineer tissues. Sustained delivery of DNA from the scaffolds may result in the transfection of a large number of cells at a localized site, leading to production of a therapeutic protein that could enhance tissue development [29]. Although gene delivery systems can be viral or non-viral, for cartilage regeneration, non-viral systems are advantageous because of their safety, low immunogenicity, and convenient application [32]. Additionally, the matrix provides the necessary 3-D space for cell proliferation, serving to maintaining elevated DNA concentrations in the cellular microenvironment, which aims to prolong gene expression and improve gene therapy effects [33]. Therefore, in this study, we evaluated a novel GAM system based on a porous chitosan scaffold embedded with HA/CS/pDNA nanoparticles encoding TGF-β1 for cartilage tissue engineering *in vitro*. The HA/CS/pDNA nanoparticles served as non-viral vectors for gene transfection in this system.

The two essential components of the GAM, the pDNA and the scaffold, could be combined in several ways. For example, naked pDNA could be combined directly with scaffold materials by adding a pDNA solution drop-wise, followed by lyophilization. Combining pDNA directly with the scaffold generates an uneven distribution of the naked pDNA on the surface and interior of the material [34], [35]. Although such a method is convenient, this combination is not stable as pDNA is unprotected from nuclease degradation, which is especially a problem in the presence of serum [10]. Therefore, in the present study, pDNA was first encapsulated in a cationic chitosan and anionic HA to form HA/CS/pDNA nanoparticles, and then nanoparticles were incorporated into chitosan scaffolds. This method is favorable since the HA/CS/pDNA nanoparticles can condense, protect, and package the pDNA for delivery into cells, as exemplified by pDNA release profiles of Figure 3.

Chitosan is a natural, biodegradable polysaccharide with demonstrated excellent biocompatibility. Multiple studies have shown that chondrocytes cultured on a chitosan-coated surface retain their characteristic round morphology and secrete chondrocyte-specific ECM constituents, such as type II collagen and proteoglycan [24], [36], [37]. Therefore, chitosan is considered a preferred material for cartilage regeneration. This was supported by our SEM analyses of the chitosan scaffolds, which clearly showed a highly porous microstructure that allowed for the transportation of nutrition to the chondrocytes through the matrix. Additionally, the micrographs demonstrated that the spherical morphology of the chondrocytes was maintained and that the cells had proliferated.

The HA/CS/pDNA nanoparticles encoding TGF-β1 were fabricated through complex coacervation, with resulting nanoparticle diameters of between 100–300 nm. These nanoparticle dimensions are suitable for chondrocyte transfection *via* endocytosis of the whole nanoparticle, or by release of the pDNA with nanoparticle degradation [16], [38]. Indeed, it has been demonstrated previously that CS/pDNA nanoparticles in the chitosan scaffold could be taken up by the periodontal ligament cells and transfected [10], and our lab showed HA/CS/pDNA nanoparticles undergoing endocytosis by chondrocytes and demonstrated their higher transfection efficiency than either CS/pDNA nanoparticles or naked plasmid DNA [19]. Although the present study provided no direct data to show the uptake of embedded HA/CS/pDNA nanoparticles in the chitosan scaffold by the chondrocytes, the data from confocal microscopy and TGF-β1-specific ELISA indicate the uptake of embedded HA/CS/pDNA nanoparticles in the chitosan scaffold by the chondrocytes. Importantly, HA in the GAM may act as a bioactive molecule where cell surface receptors for HA (such as CD44) facilitate chondrocyte-scaffold interactions, thereby promoting adhesion, proliferation, and transfection of chondrocytes [19], [39]. Although the transfection efficiency of HA/CS/pDNA nanoparticles does not reach the same high level as Lipofectamine™ 2000 [19], the predominant biocompatibility and sustained pDNA release properties of HA/CS/pDNA nanoparticles make them a promising gene transfer carrier for chondrocytes [40]. Moreover, as Yamane et al. reported, introducing HA to the chitosan materials may help to significantly increase the material properties of the scaffold because ionic interaction readily forms a tight bond between two molecules [20]. As chitosan is a cationic polysaccharide consisting of glucosamine residues and HA has anionic behavior, a tight bond between both molecules is expected.

The pDNA release characteristics of nanoparticles suggested that the GAM embedded with HA/CS/pDNA nanparticles was a well controlled system capable of releasing pDNA for a lengthy period of more than 120 days. As expected, pDNA was released from either HA/CS/pDNA nanoparticles or nanoparticle-embedded GAM in a sustained fashion. Both isolated nanoparticles and GAM release profiles were characterized by an initial small burst release of about 10–20% of the pDNA in the first 24 h, followed by a much slower release at a constant rate. The initial burst release may be the result of untrapped naked pDNA that has attached to the nanoparticle surface and diffuses easily into the medium [41]. The slower subsequent degradation of nanoparticles and scaffolds resulted in the loss of polymer mass and a slower pDNA release phase [42]. This type of release is a major reason that the GAM embedded with HA/CS/pDNA nanoparticles displays a significantly more controlled delivery than GAM embedding naked pDNA. For GAM embedded with naked pDNA, pDNA level rises quickly and then decline suddenly as the pDNA is cleared or degraded, which does not support long-term gene therapy [10]. In the present study, though the initial burst release was significant, the majority of pDNA was cumulatively released from GAM and HA/CS/pDNA nanoparticles under a longer and more sustained period, as indicated by the fact that at 120 days release was (39.21±0.22)% and (61.47±0.59)%, for GAM and nanoparticles, respectively. These observations were consistent with previous reports [25]. As shown in Figure 3, it was obvious that pDNA release was much faster in HA/CS/pDNA nanoparticles than that in GAM. This may be due to increased resistance from chitosan when pDNA is released from the GAM, as compared with that from the nanoparticles. Moreover, in GAM, the DNA released from the nanoparticles may complex again with the cationic amino group (–NH_2_) of the chitosan scaffold, thereby limiting the free pDNA released to the medium. Even after four months, the pDNA released from the GAM was still maintained at an adequate level for cartilage regeneration.

Transfection efficiency and the expression level of TGF-β1 are two highly important characteristics of the GAM. In this study, for cells cultured with the GAM, confocal laser scanning microscopy was used to detect the GFP-positive transfected cells, while ELISA was used to determine how much TGF-β1 was produced by chondrocytes. Confocal microscopy demonstrated that GFP-positive transfected cell clusters present 7 days after culture in the two types of GAM, and as expected, no transfection was detected in the control group that was free of DNA. This indicates that HA/CS/pDNA nanoparticles successfully transfected chondrocytes cultured in the 3-D GAM.

Expression of TGF-β1 in cells cultured with GAM embedding HA/CS/pTGF-β1 nanoparticles was significantly higher than that of the two control groups during 21 days of culture. According to the ELISA results, TGF-β1 expression could be detected after three days of culture, reaching its highest level on the sixth day. This may be due to the fact that primary chondrocytes have a relatively static growth period until they achieve integration with the architecture of their microenvironments and only begin to express the target protein after integration. Moreover, in order to function as viable gene vectors, the HA/CS/pDNA nanoparticles must escape endosome–lysosome enzymatic dissociation after endocytosis by chondrocytes, and upon entry into the cell be degraded by a polysaccharide enzyme before the pDNA can be gradually released and enter into the nucleus. This time-dependent intercellular process might explain why the expression of foreign transfected genes remained low during the first few days of culture, with the transfection efficiency increasing after prolonged times [19].

It is well known that DNA (especially DNA containing CpG sequences) can activate cells [9]. Therefore, a control group using empty pDNA is necessary to estimate the influence of TGF-β1 expression, in addition to pDNA. In the present study, we used the same plasmid vector containing an EGFP sequence as the control plasmid group for the cell proliferation assay, confocal laser scanning microscopy, ELISA, histology, and GAG assay. The results of all assays showed that chondrocyte proliferation was indeed activated by the GAM containing the empty plasmid in comparison to the DNA-free chitosan scaffold containing no nanoparticles, and these data were qualitatively confirmed by SEM imaging (Figure 4) and histological analysis (Figure 11). It is also possible that the addition of nanoparticles to the scaffold enhanced its specific surface area, leading to increased cell seeding efficiency and hence the differences found between the scaffolds after culturing. However, the ELISA assay showed only a small increase in protein expression in the empty nanoparticle GAM group, in comparison to that of the chitosan scaffold containing no nanoparticles. Protein expression in both groups remained significantly lower than that in the pTGF-β1-activated group.

To assess maintenance of the chondrocytic phenotype, GAG production within these constructs was examined, since this molecule is an important component of cartilage ECM. As shown in Figure 11, no significant differences in GAG content per chondrocyte were observed over the culture period. Thus, chondrocytes in all three formulations appeared to maintain a constant level of GAG production, which was consistent with previous studies [6]. Under histological observation, cells within these constructs also appeared to maintain their intrinsic round morphology, providing further evidence of phenotype preservation. Overall GAG content per sample appeared to increase significantly from day 3 values with all three constructs. However, since no significant differences were observed in GAG per cell (DNA) values, these results suggest that enhancement in ECM production was primarily due to increased cellularity. GAG within these constructs appear to be concentrated in the pericellular region, as shown by the toluidine blue staining surrounding the chondrocytes (Figure 7).

Although the biological activity of the TGF-β1-encoding pDNA loaded in the HA/CS/pDNA nanoparticles was not quantitatively confirmed in this study, the results indicate activity was preserved during nanoparticle and GAM preparation, and during subsequent cell culture. TGF-β1 is known to promote chondrocyte proliferation, as demonstrated by several previous studies [5], [6], [8]. It has also been reported that TGF-β1 can enhance proteoglycan deposition, and that its biological activity *in vitro* can be affected by a multitude of factors, such as the state of cellular differentiation, the growth conditions, the ingredients of culture medium (especially the concentration of fetal bovine serum), and the presence of other growth factors [8]. However, most of these previous studies showed that the absolute amount of GAG was significantly higher in the TGF-β1-supplemented scaffolds than that in the control groups. While cell-doubling time is an important measure of proliferative activity, the 3D culture system with cells adhered to the scaffolds used in our study complicated (and precluded) this type of analysis; yet, the fact that the cell culture medium was carefully made to exclude any potentially influential factors (such as growth factors or fetal bovine serum) helped to ensure the validity of our observations. Both production of cartilage-specific ECM and chondrocyte proliferation were significantly higher in the GAM embedded with HA/CS/pTGF-β1 nanoparticles, in comparison to the control groups with empty plasmids or chitosan-only scaffolds. Furthermore, IHC revealed that synthesis of type II collagen was most pronounced in cells cultured in the GAM embedded with the HA/CS/pTGF-β1 nanoparticles, as compared to the two control cultures. Moreover, the results of the biochemical assay also verified that TGF-β1-activated GAM significantly increased the cell DNA amount, as compared with both control groups, suggesting that this novel GAM significantly promoted chondrocyte proliferation. The results of confocal laser scanning microscope observation and ELISA also demonstrate that HA/CS/pDNA nanoparticles could successfully transfect chondrocytes cultured in the 3-D GAM and that transfected cells secrete TGF-β1. These comprehensive results indicate that GAM embedding HA/CS/pDNA nanoparticles encoding TGF-β1 can serve as an enhanced cartilage tissue engineering scaffold and sustained gene delivery system, providing a nonviral vector for pDNA delivery and an effective scaffold material for chondrocyte adherence and proliferation.

Although we proved that HA/CS/pTGF-β1 nanoparticles effectively transfected chondrocytes and produced higher levels of TGF-β1 during the culture period through confocal laser scanning microscope observation and ELISA etc, the main limitation of this study is that we did not get an accurate transfection efficiency, nor verify its effectiveness *in vivo*. Further studies are needed to fully address the transfection efficiency in the GAM, its influencing factors, and its application *in vivo*.

### Conclusions

The present study designed and evaluated a novel GAM composed of a chitosan scaffold with embedded HA/CS/pDNA nanoparticles encoding TGF-β1 for cartilage tissue engineering applications. Delivery of pDNA from the GAM was indicated to be steady and sustained for more than 120 days, which was even slower than that of HA/CS/pDNA nanoparticles. Chondrocytes cultured with GAM showed increased TGF-β1 expression, enhanced proliferation, and did not appear to affect phenotypic expression of ECM molecules, such as GAG. In conclusion, a GAM consisting of a chitosan scaffold embedding HA/CS/pDNA nanoparticles inducing expression of TGF-β1 may be a promising system for enhancing *in vitro* cartilage tissue engineering.

## Supporting Information

Figure S1 **Synthesis of chitosan-HA.**
(TIF)

Figure S2 **Confocal microscopic images of chondrocytes treated with FITC-DNA nanoparticles.**
(TIF)

Figure S3 **Histology (H&E staining) of chondrocytes seeded in three different scaffold types.**
(TIF)

Video S1 **3D Confocal laser scanning of pEGFP in 7d.**
(MPEG)

Video S2 **3D Confocal laser scanning of pTGF in 7d.**
(MPEG)

Video S3 **3D Confocal laser scanning of pEGFP in 21d.**
(MPEG)

Video S4 **3D Confocal laser scanning of pTGF in 21d.**
(MPEG)
