# Supplementary figures and images for: Porous Chitosan Scaffolds with Embedded Hyaluronic Acid/Chitosan/Plasmid-DNA Nanoparticles Encoding TGF-β1 Induce DNA Controlled Release, Transfected Chondrocytes, and Promoted Cell Proliferation
Source: PLoS One. 2013 Jul 23;8(7):e69950. doi: 10.1371/journal.pone.0069950 (PMC3720934; doi:10.1371/journal.pone.0069950)

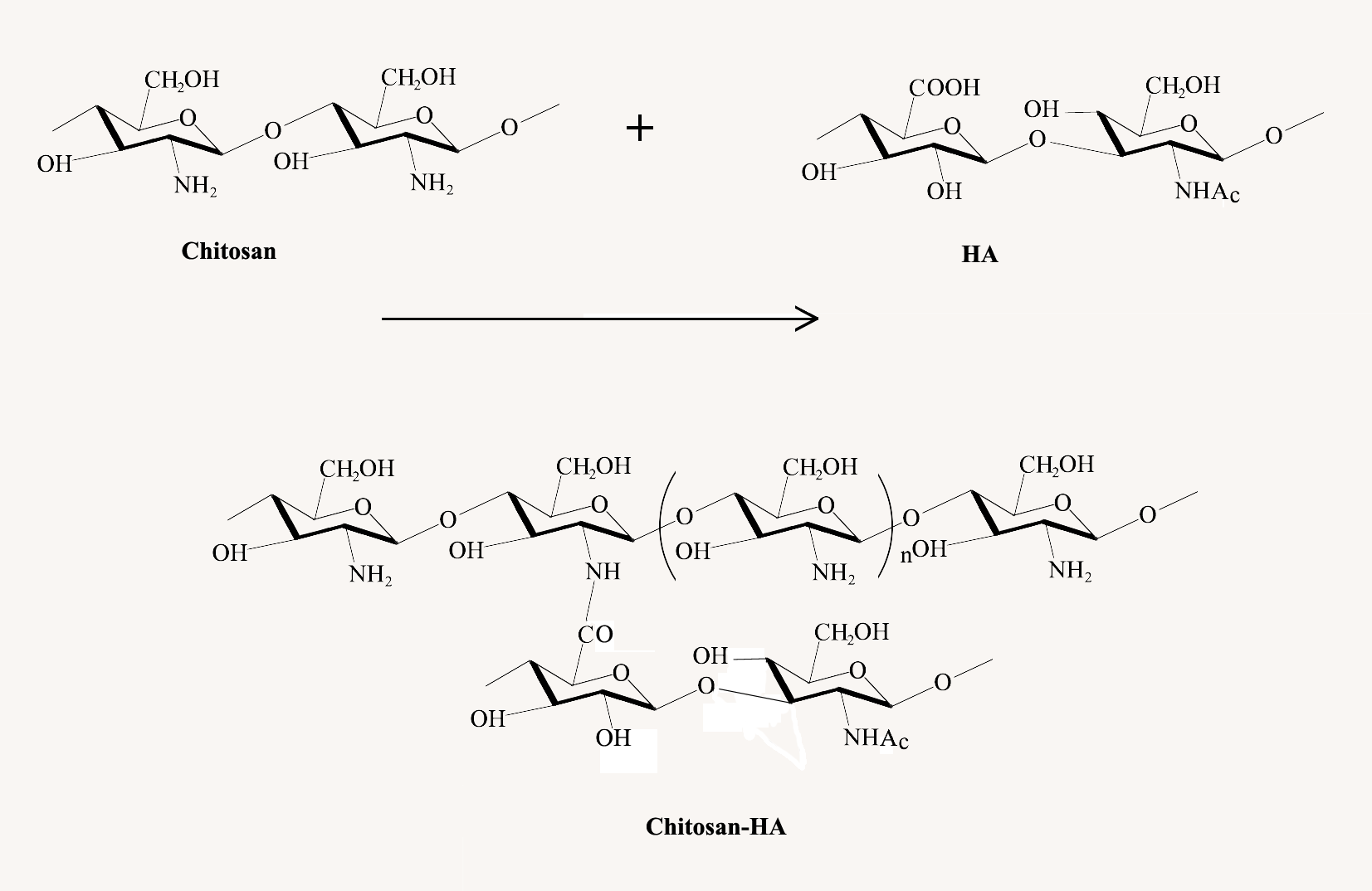

Supplement: Figure S1 — Synthesis of chitosan-HA. (TIF) [file pone.0069950.s001.tif]

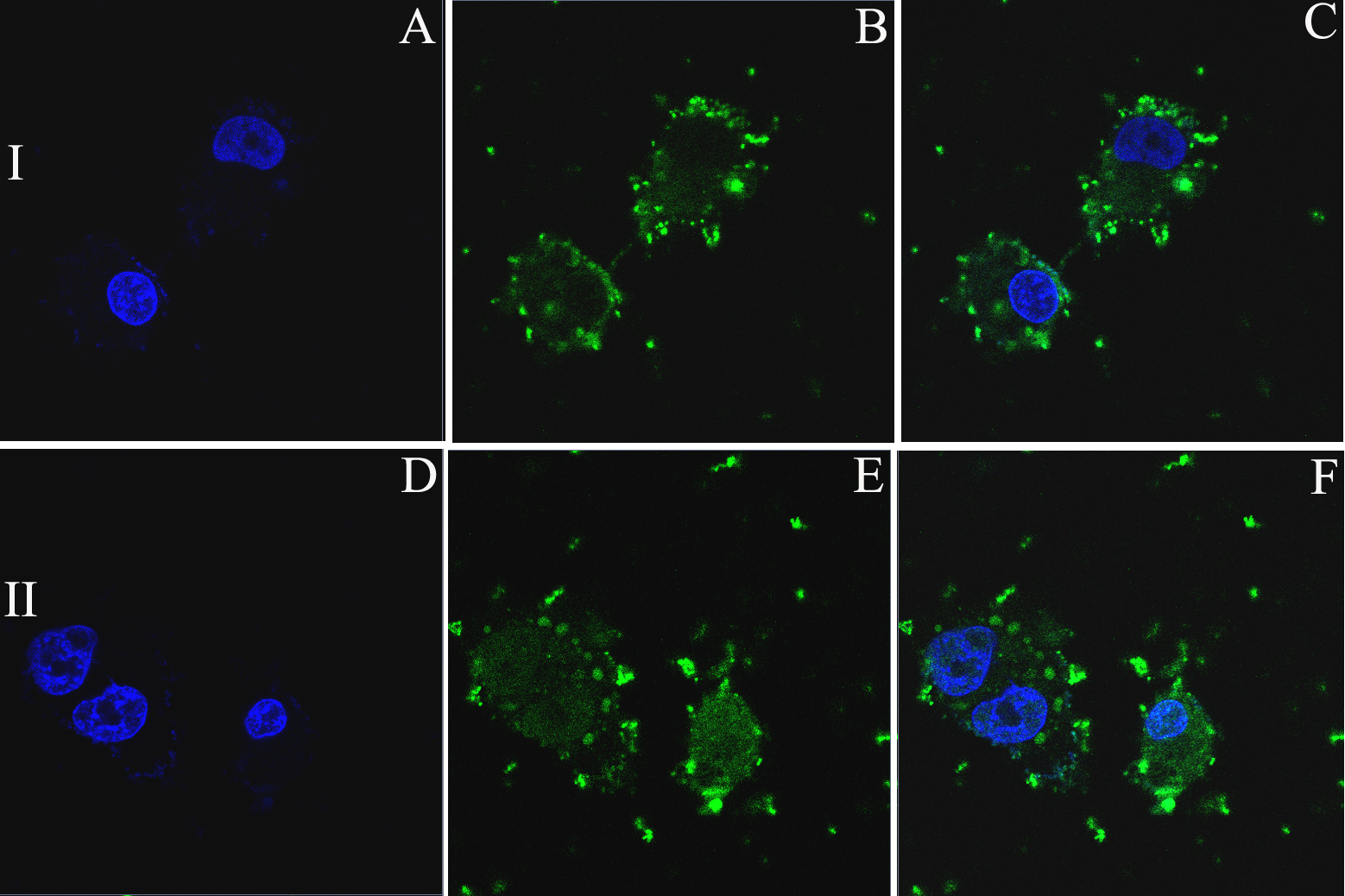

Supplement: Figure S2 — Confocal microscopic images of chondrocytes treated with FITC-DNA nanoparticles. (TIF) [file pone.0069950.s002.tif]

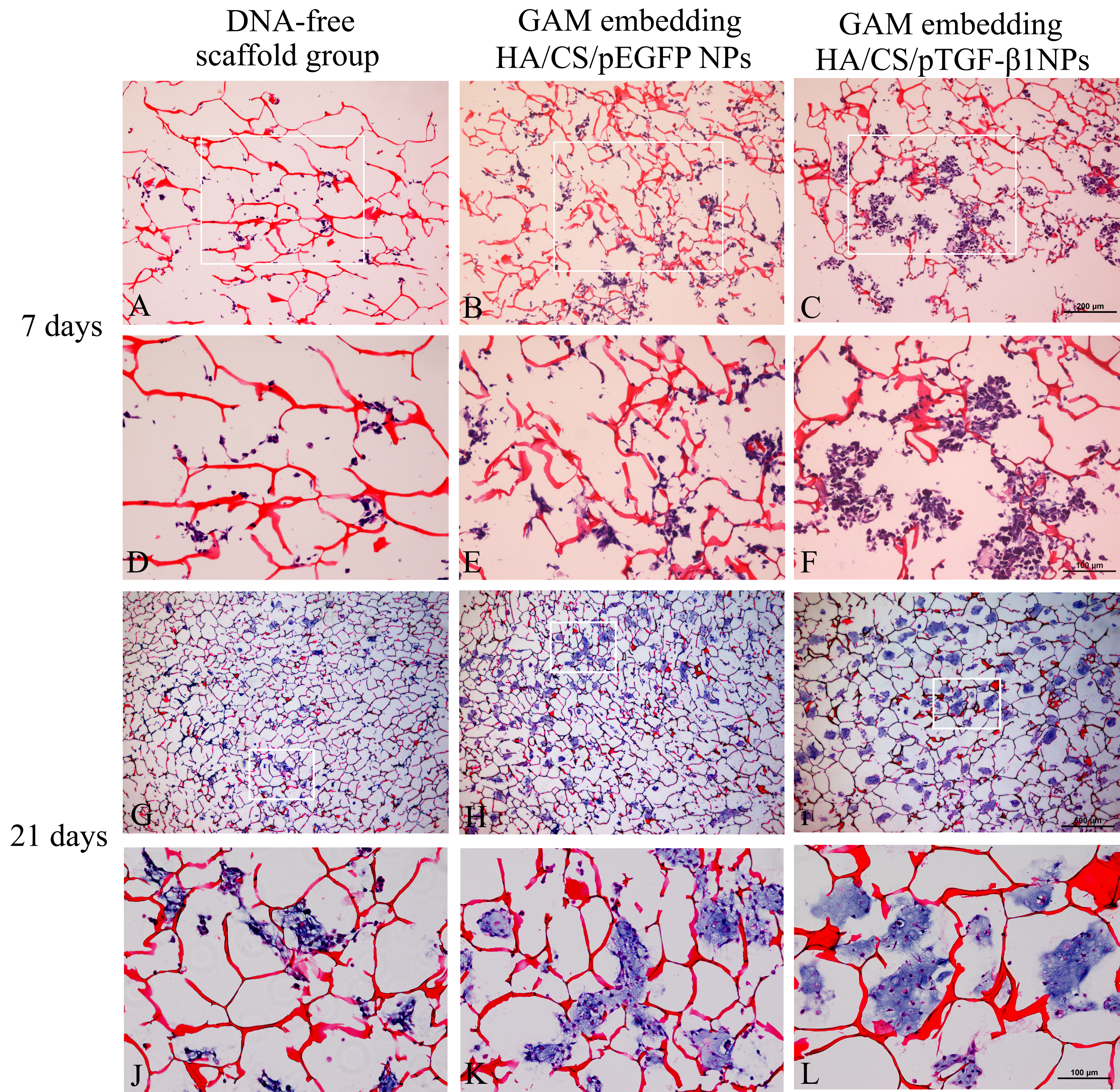

Supplement: Figure S3 — Histology (H&E staining) of chondrocytes seeded in three different scaffold types. (TIF) [file pone.0069950.s003.tif]
